# Development and Validation of a Mixed Reality Configuration of a Simulator for a Minimally Invasive Spine Surgery Using the Workspace of a Haptic Device and Simulator Users

**DOI:** 10.1155/2021/2435126

**Published:** 2021-12-31

**Authors:** Sneha Patel, Sami Alkadri, Mark Driscoll

**Affiliations:** Department of Mechanical Engineering, McGill University, MacDonald Engineering Building, 817 Rue Sherbrooke Ouest #270, Montréal, Québec, Canada H3A 0C3

## Abstract

Most surgical simulators leverage virtual or bench models to simulate reality. This study proposes and validates a method for workspace configuration of a surgical simulator which utilizes a haptic device for interaction with a virtual model and a bench model to provide additional tactile feedback based on planned surgical manoeuvers. Numerical analyses were completed to determine the workspace and position of a haptic device, relative to the bench model, used in the surgical simulator, and the determined configuration was validated using device limitations and user data from surgical and nonsurgical users. For the validation, surgeons performed an identical surgery on a cadaver prior to using the simulator, and their trajectories were then compared to the determined workspace for the haptic device. The configuration of the simulator was determined appropriate through workspace analysis and the collected user trajectories. Statistical analyses suggest differences in trajectories between the participating surgeons which were not affected by the imposed haptic workspace. This study, therefore, demonstrates a method to optimally position a haptic device with respect to a bench model while meeting the manoeuverability needs of a surgical procedure. The validation method identified workspace position and user trajectory towards ideal configuration of a mixed reality simulator.

## 1. Introduction

Spinal surgeries require intricate motor skills due to the proximity of the spine to neurological components and potential adjacent vascularization. Improper practice of surgical techniques leads to serious complications; for example, neural injuries, pulmonary embolus, neurological deficit, and infection within the operating area [1, 2]. The smaller incision used with minimally invasive (MI) surgeries is beneficial for patients as it limits the postoperative pain, reduces hospital stay, reduces blood loss, and shortens recovery time [3–5]. However, with MI procedures, the likelihood of serious complications increases due to the constraints imposed by the surgical environments. Further, despite the patient benefits, MI procedures are associated with limitations for surgeons, including a 2-dimensional view of the operating area from a video camera, hindered hand-eye coordination, tremor in the surgical tools, fulcrum effect of surgical tools about the incision point, and a need for dexterity of both hands [6]. The fulcrum effect refers to the tool moving in the opposite direction in the surgical area when moved in a specific direction by the surgeon outside the incision and is based on the constraints set forth by the surgical port or the incision [7, 8].

Many teaching hospitals acknowledge the risks conventional training methods have due to on patient training and the steep learning curve associated with MI procedures [9]. Therefore, researchers suggest the use of inanimate training systems to teach surgical skills and tasks, for example, box trainers or virtual reality (VR) training systems [10, 11]. VR systems are gaining popularity for surgical training due to recent technological developments and the success of similar training systems for pilot training [12, 13].

The most important technological development towards successful surgical training systems is haptic devices, which allow users to interact with the VR environment and experience force feedback corresponding to manipulation of objects within said space. Haptic information allows the user to experience the virtual environment using the sense of touch through force or tactile feedback [14, 15]. The feedback adds to the realism of the training system and better represents what a surgeon would experience in an operating room [16]. Haptic information is essential towards the development of fine motor skills, such as those required in a MI spine surgery. Haptics allows the user to interact with the VR environment accurately based on the force feedback experienced while virtually manipulating various tissues in the surgical areas [17].

Present haptic devices collect position data of the end effector (EE) from the user and convert them into force output based on the interactions within the simulations. The force feedback is dependent on where the EE is in the VR environment and whether a collision is detected between the surgical tools, which are manipulated using the haptic device using analog tools modelled on both ends of the EE, and other objects in the virtual environment [18]. Haptic devices can also be used as tools to assess users' performance and changes in motor skills, through the manipulation of the EE, both of which are vital for determining the preparedness of the user to perform the surgical technique on a patient [19–21].

Both tactile and force feedback are important for realistic haptics or feel during a surgery, as is suggested by multiple studies in surgical robotics [22, 23]. Two types of trainers are most commonly used to simulate spine procedures and train residents in these complicated procedures, VR [24, 25], and physical trainers [26, 27]. Most commonly three-dimensional printed models of patient-specific vertebral bodies are used to train surgeons in the required skills for spine surgery [26, 28–30]. More recently, a few mixed reality simulators have been presented in literature for spine surgery training, which use the virtual environment to model the physiological responses while the physical model of the surgical area supports the haptic device towards providing realistic feedback [31, 32]. These simulators aim to incorporate the best aspects of both types of trainers for a spine fusion procedure as mentioned in a study by Coelho and Defino, which presents the potential of such systems [33]. As this approach is fairly new for training surgeons in the field of spine surgery, there lacks an objectively validated workflow to define the optimal configuration of the simulator components which enables the required surgical workspace and thus potentially foster motor skills to successfully complete the procedure. Furthermore, to the authors' knowledge, there are no mixed reality trainers, which provide training on all tasks within a spine fusion technique, i.e., Transforaminal Lumbar Interbody Fusion. The proposed training system in this study will use a mixed reality system, that is, (1) a haptic device to provide force feedback and (2) a bench model of a section of the human spine for additional and complementary tactile feedback. The bench model plays two key roles, (1) it provides rigid body interactions (i.e., bone and surgical tool) which is difficult to model in haptic devices [34–36] and (2) provides a model of the back so that the surgeons could interact with it while stabilizing the cylindrical port through which the surgery is performed. The integration of the two requires an analysis of the kinematics of the haptic device to determine the optimal position of the device with respect to the bench model while understanding the needs of the surgical procedure and the constraints imposed by simulator components. To successfully integrate the bench model and haptic device, the device and surgical procedure workspaces must be determined. Using these workspaces, various configurations are calculated to determine the optimal position for the haptic device with respect to the bench model.

This study is aimed at developing and evaluating a method that leverages forward kinematics of a haptic device, Entact W3D (Guelph, Ontario, Canada), to determine the optimal position of the device considering its own hardware limitations and the anticipated minimally invasive surgical manoeuvers. This method was validated using surgical workspace and data from expert surgeons and may be adapted for other future mixed reality surgical simulators.

## 2. Materials and Methods

### 2.1. Computing the Haptic Device Workspace

The workspace of the haptic device was calculated using forward kinematics to obtain the position and orientation of the EE by manipulating the joint-specific variables and parameters. These values were calculated using the general homogeneous transformation matrix, *T*, and specifying the joint variables, *θ*, at any instance of the haptic device. Using this information, the workspace of the manipulator of the haptic device was constructed.

This study determined the workspace through a numerical approach using the results from the forward kinematics and the homogeneous transformation matrices. Possible configurations of the manipulator were then calculated using nested loops. Modeling the haptic device as a robotic arm the Denavit-Hartenberg (DH) table (Table 1) was constructed for the Entact W3D haptic device. Entact W3D allows motion in six degrees of freedom, force feedback along three degrees of freedom, and with gravity compensation. The range of each joint variable (*θ*_*i*_) was dictated by the haptic device. Using the parameters of the respective DH table, the kinematics of the haptic device was analysed using techniques outlined by Craig [37].

### 2.2. Numerical Validation of Computed Haptic Device Workspace

Based on the results in the previous section, a validation was completed by comparing it to the geometrical workspace. The calculated workspace was compared to the geometrical workspace or the computer-aided design (CAD) workspace based on the physical design of the haptic device. These CADs were provided by the haptic device manufacturer and were compared to the volume of the workspace determined in this study. The CAD workspace, as provided by the manufacturer, is based on the design of the device, including the case design for the haptic device and the range of motion of its joints.

### 2.3. Determination of Position of Haptic Device Relative to Surgical Bench Model

Based on the validation of the computational workspace and requirements of the mixed reality simulator, a study was developed to determine the position of the haptic device with respect to the bench model, a partial physical representation of the surgical area. Constraints were identified with respect to the physical boundaries of the components within the simulator and the surgical procedure. This required an analysis of the surgical procedure of interest and factors that affect the workspace required to naturally move tools in the procedure and the components within the simulator which limit the movement of the EE through the simulator. These factors were considered when calculating the inverse kinematics of the haptic device to determine the configuration of the haptic device within the simulator.

**Figure 1:**
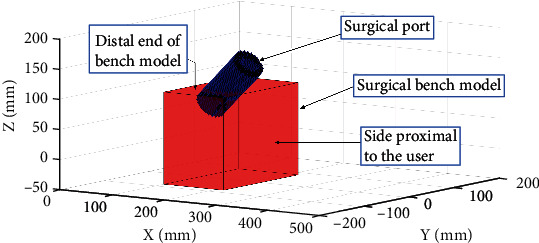
Cylindrical volume, the surgical port, of operation within the model of the human back, labelled surgical bench model.

Some constraints associated with the simulator's components were also considered during this validation study. For example, the simulator would need to be designed for both left- and right-handed users. Also, a major constraint of the surgical procedure is that the MI spinal fusion is performed through a small cylindrical port with a diameter of 15 mm. Due to the restrictive diameter of the port and the larger size of the haptic device's EE, the EE would never enter the port during the simulation. As seen in Figure 1, the port was placed on the bench model to reflect the maximum rotation of the port with respect to the virtual patient's spine in the MI procedure of interest. A requirement of 100 mm was set for the displacement of the EE along the path of the port to perform the expected manoeuvers using the surgical tools without any restrictions. This applies to tasks that would be performed through the port in a spinal fusion procedure; for example, discectomy, a task which requires all six degrees of freedom, albeit confined.

This study focused on the development of a training simulator for a MI spinal fusion procedure, which uses a posterior approach to limit morbidity associated with the procedure [38]. As the MI procedure has a small access point from the skin, the surgeons typically work through a surgical port placed at an incision, 30-50 mm from the midline of the back in order to gain access to the pedicles and the facets of interest [39–41]. The desired access requires the port to be placed at an approximate angle range of 45-90° with respect to the skin of the patient. For a spinal fusion procedure, the user must be able to perform both translation and rotation manoeuvers of the surgical tools within the port. The port's rotation was considered during the development of the required workspace as the lower end of the range would require a larger workspace from the haptic device.

The port was considered in the kinematic study. The surgical bench model and port were plotted in Figure 1 using MATLAB 2018a (Natick, Massachusetts, United States). This plot, coupled with the plotted workspace, was created to better determine the placement and orientation of the haptic device relative to the bench model of the mixed reality simulator. This provided a visualization of the problem at hand. It is to be noted that the port was immersed in and extended beyond the top surface of the bench model to mimic the port placement during a surgical procedure. As the haptic device's EE would never enter the port the perception of the surgical tools being in the surgical area would be provided through force feedback and interactions with a distal tip of the surgical tool connected to the EE.

### 2.4. Validation of Position of Haptic Device Relative to Surgical Bench Model by Analysing User Data

Lastly, the validation results were used to develop a user-based study, completed at McGill University and Depuy Synthes. The objective of this study was to collect data from users in a novel posterior approach MI spinal fusion procedure during the initial access step to reach the intervertebral disc (IVD) using a probe and during facetectomy using a surgical burr. This experiment was approved by McGill's Institutional Review Board (IRB approval number: A03-M15-20A). The study that was conducted at McGill University included three users with no surgical/medical background. The study that was completed at Depuy Synthes comprised of seven expert surgeons who performed the entire procedure on cadavers using the actual surgical tools immediately prior to testing the simulator. This step was adopted to ensure surgeons were familiar with the procedure in an effort to enable fluid movement when using the devised simulator. The surgeons' experience in spinal fusion ranged from 3 to 13 years. While using the simulator, user trajectories were collected and then superimposed on the workspace that was developed using the kinematics of the device. The initial step of the surgical procedure was of great interest in this study because the surgeon had a large workspace as they determined the placement of the surgical port in order to access the surgical area through the port. A grad student acted as a surgical nurse, attaching, and handing over the relevant model of the surgical tools at the EE of the haptic device, as the surgeons worked through the simulator.

**Figure 2:**
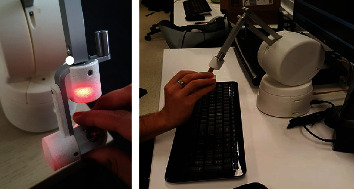
Example of haptic device use for workspace determination of the surgical simulator.

Figure 2 demonstrates users manipulating the EE of the haptic device in the simulator. The device's configurations were limited to changes along the *z* direction or the height of the haptic device with respect to the bench model. The height at which the haptic device should be placed with respect to the bench model was determined based on the results of the workspace calculation and the workspace determined by surgeon trajectories in the simulator.

Based on the collected user trajectory data, Kruskal-Wallis tests were completed to further support the finalized configuration of the mixed reality simulator.

## 3. Results and Discussion

### 3.1. Computing Haptic Device Workspace

**Figure 3:**
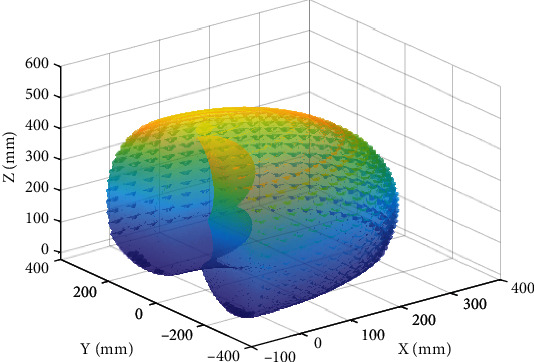
MATLAB generated workspace of the haptic device.

Figure 3 presents a plot of the workspace for the haptic device. The volume is comparable in shape to the geometrical representation provided by the device manufacturer. There are a few discrepancies between the two models as the joints are constrained by the physical structure of the haptic device despite the range of motion permitted by the joints of the robotic arm.

### 3.2. Validation of Computed Haptic Device Workspace

**Figure 4:**
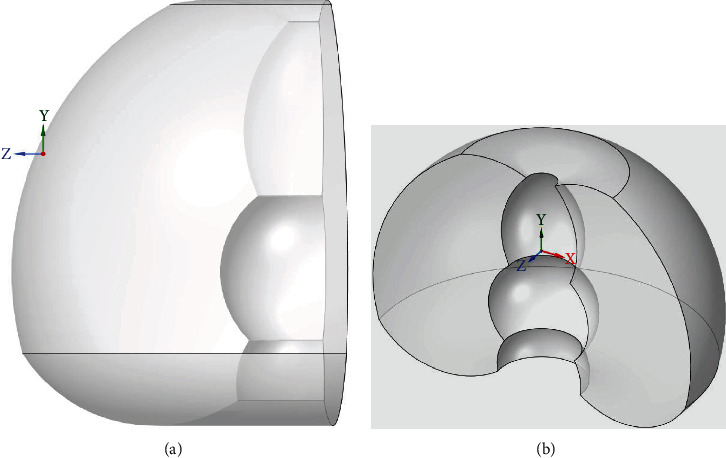
The workspace CAD from the haptic device manufacturer (a) three-dimensional view and (b) side view.

Figure 4 presents an approximation of the workspace CAD that was provided for this study by the device manufacturer. The volume of the CAD is 6.37 × 10^7^ mm^3^ whereas the workspace that was developed using the kinematics of the haptic device has a volume of 6.44 × 10^7^mm^3^. Therefore, the calculated workspace is approximately 2% larger than the provided CAD workspace.

The forward kinematic study allowed for the development of the workspace of the haptic device where in this study, the focus was on Entact's W3D. This workspace was compared with the workspace of the surgical procedure with respect to the entry point and the surgical tools that are modelled by the simulator. The workspace developed mathematically is 2% larger which could be attributed to not accounting for the physical body of the haptic device. This is a negligible error in the calculation with respect to the geometrical model that is provided by the manufacturer since the workspace of the haptic device compensates for the area of the surgical procedure without any restrictions. Examination of the haptic device demonstrated that despite the allowed range of motion of the joints, the joints are limited by the physical structure of the haptic device, including the body of the haptic device and the encoders on three joints.

### 3.3. Position of Haptic Device Relative to Surgical Bench Model

**Figure 5:**
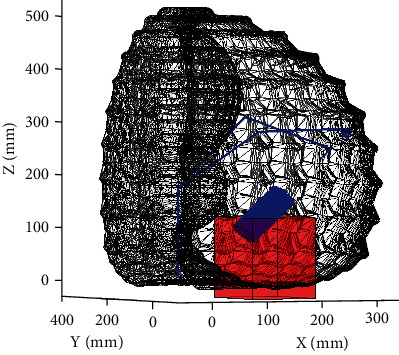
Intersection of the haptic device's workspace with the bench spine model (black—frame of device workspace, red rectangular prism—bench model, blue cylinder—port, blue lines—haptic arm representation).

The plot in Figure 5 placed the workspace of the Entact haptic device with respect to the bench model of the human spine. This plot demonstrates that the workspace of the haptic device supports the workspace of the surgical bench model that would be used in the simulator because the workspace of the haptic device encapsulates the workspace of the MI spinal fusion procedure within the simulator and the required movements of the robotic arm.

**Figure 6:**
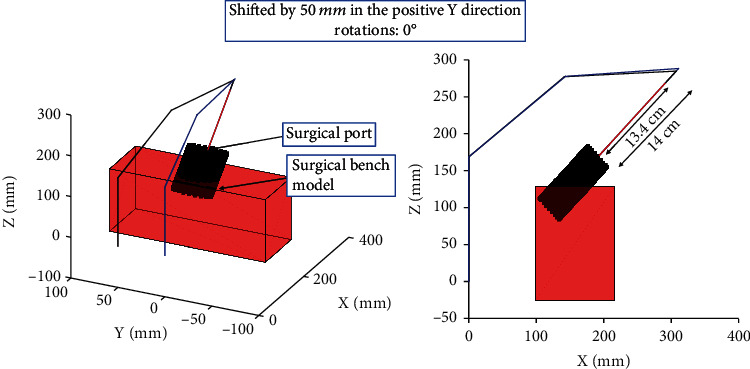
The effect of translating the base of the device from the starting position (middle of the bench model) towards the positive *y* direction. This observed a translation of 50 mm.

**Figure 7:**
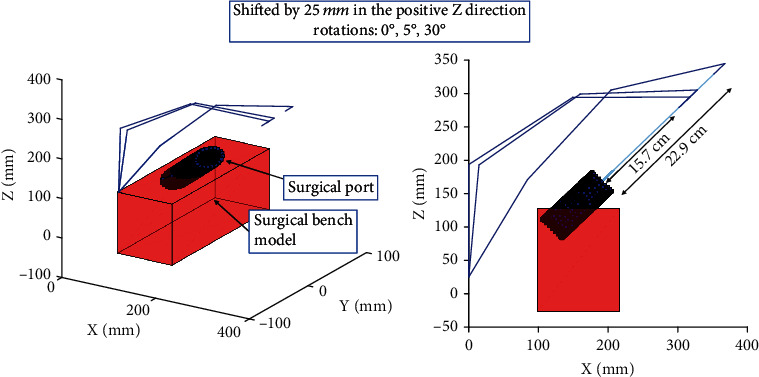
The effect of translating and rotating the base of the device from the starting position towards the positive *Z* direction. A translation of 25 mm is tested with three rotations, 0°, 5°, and 30°.

Using inverse kinematics and the constraints of the simulator, the configuration of the device and its manipulator was determined. Forward kinematics was then used to plot the configuration given by the inverse kinematic solution.  Figure 6 illustrates the effect of translating the haptic device along the *y* axis of the bench model where the possible translation of the EE along the path of the port decreased by 6 mm. Conversely, Figure 7 demonstrates the effect of elevating the device and rotating its base, which shows a drastic change in the EE's range of motion along the path of the surgical port. The rotation of the base of the haptic device provides a larger workspace but limits the rotation of long surgical tools, typical of those used in MI surgeries, which will be attached to the EE of the haptic device. Elevating the device increased the maximum translation of the EE along the trajectory of the port by 17 mm. This therefore allows the user to perform motions along the trajectory of the surgical port as required in a MI spinal fusion.

Based on the inverse kinematic analysis and the constraints of the simulator, the optimal placement of the haptic device with respect to the bench model was determined. Due to the constraint of usability for users with either hand as the dominant hand, the device was placed behind the bench model towards the center of the surgical area. The device was also placed 25 mm above the base of the bench model, and its center was 100 mm away from the bench model representation (dimension 160 mm × 160 mm) to increase the workspace along the path of the port.

The inverse kinematics of the device was used to move the EE of the haptic device and determine the travel path that is permitted by the device's workspace with respect to the trajectory of the surgical port. The inverse kinematic study therefore demonstrated that the device's manipulator can extend up to 157 mm from the tip of the port, thus, allowing the required manoeuvrability for the surgical tools. The user trajectory data further supported the use of a platform to raise the haptic device to provide the users with a larger workspace based on the needs of the surgical procedure. It demonstrated that the EE could be moved in the simulator without leaving the workspace. The users' trajectory remained within the ideal area and did not require overextension of the arms of the device.

**Figure 8:**
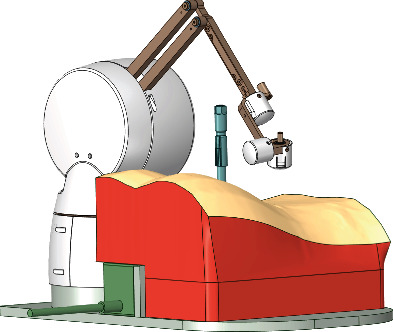
The bench model design determined by the kinematic study performed in this study and the constraints set for the design.

Based on the results using the forward kinematics and inverse kinematics, an appropriate benchtop configuration was determined. This configuration is illustrated in Figure 8, which includes the haptic device, the bench model (The Chamberlain Group, Great Barrington, Massachusetts, United States), three dimensional printed vertebral bodies, and a source for a motion tracking system, Polhemus Patriot (Colchester, Vermont, United States). A proprietary game engine was used from our partners CAE Inc. (Montreal, Quebec, Canada) which was linked to the Entact haptic device using API developed in Microsoft Visual Studio 2015 (Redmond, Washington, United States) in C++. As was determined in Figure 7, increasing the platform height by 25 mm, for the haptic device, provides the user with the predetermined, required space along the path of the port. The configuration thus applied this modification to position of the haptic device with respect to the bench model and other components of the simulator.

### 3.4. Validation of Haptic Device Placement

**Figure 9:**
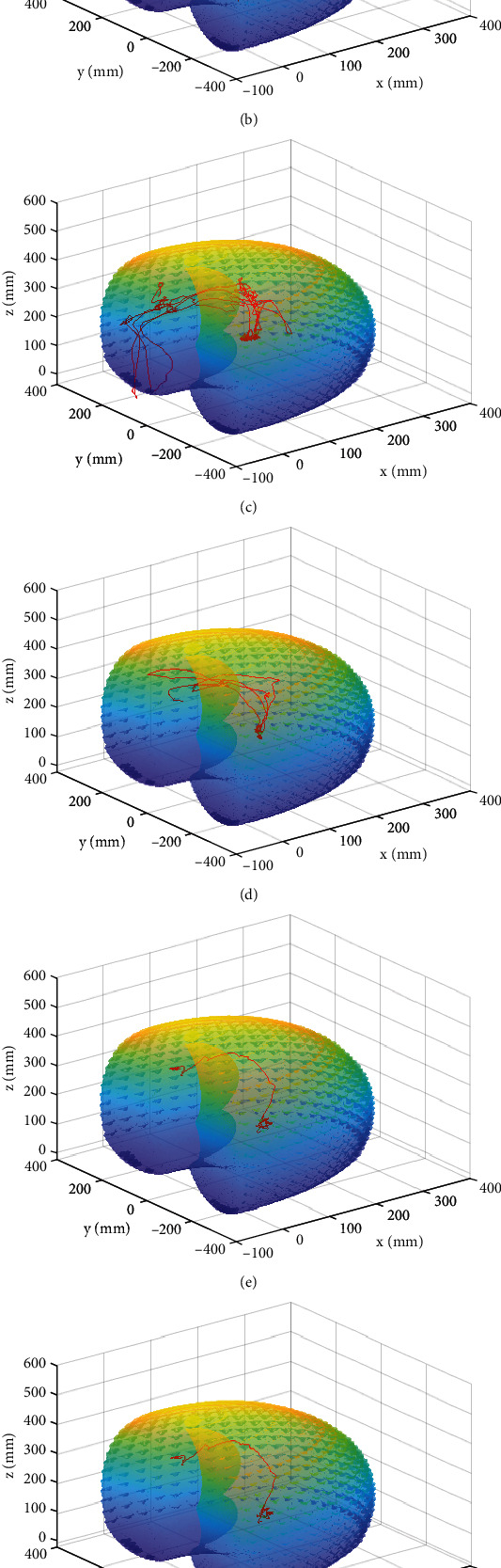
Illustrates the trajectories of users on the simulator overlaid by the haptic device's workspace. (a) shows the trajectory of a surgeon using the simulator for the first time, and (b) shows the trajectory of a user with no medical background but familiarity with the simulator. Both of these trajectories were collected when the user was gaining access to the area of interest. (c) and (d) show the trajectories of two surgeons during the facetectomy which was completed through a cylindrical, surgical port. (e)–(g) are the trajectory of a surgeons overlaid on the workspace of the haptic device while considering different heights for the placement of the device, (e) same height as the bench model, (f) 10 mm above the bench model, and (g) 25 mm above the bench model, respectively.

The plots in Figure 9 compared samples collected from different users in their attempt to reach the IVD (a and b), a task which would require the largest workspace in an MI spinal fusion procedure, and (c and d) during facetectomy which was completed through the surgical port. Further, in Figures 9(e)–9(g), the trajectory of a single user was overlaid on the workspace of the haptic device at different heights with respect to the bench model to assess the ideal configuration. In each case, the haptic device's workspace encompassed the movements of the user in different configurations of the bench model setup thus validating the placement in regard to encompassing surgical manoeuvres. Further assessment sought to explore any trends in this movement between users.

Trajectory volumes were calculated for the two tasks of interest in this study. For the access to IVD step and the facetectomy through the surgical port, a cone volume and cylindrical volume of the trajectories were calculated, respectively. A cone volume for the initial task was calculated because the surrounding soft tissue would provide enough resistance to form a cone-shaped workspace. On the other hand, the surgical port's shape provided a hollow cylindrical workspace, and the cylindrical volume was calculated for the task completed through the port. The average cone volume was 5.82 × 10^5^mm^3^ with a standard deviation of 3.44 × 10^5^mm^3^. The average cylindrical volume was 5.02 × 10^5^mm^3^ with a standard deviation of 7.21 × 10^5^mm^3^. Much like the translations measured in Figures 6 and 7, the translations along the port during facetectomy were an average of 142.5 mm with a standard deviation of 478.4 mm. The trajectory and volume calculations data associated with tool changes and handing over to surgeons were disregarded as it is not considered a part of the simulation or the surgical procedure.

The statistical analyses completed for the user data are presented in Table 2. The nonparametric analysis, Kruskal-Wallis (nonparametric ANOVA), was used in this study. As can be seen in Table 2, the *p* value for the comparison of the surgeons' trajectories in the simulator was very low, suggesting that the trajectories of the medically trained users were significantly different. On the contrary, the Kruskal-Wallis analysis of the trajectory volumes of users familiar with the simulator and no medical background suggested a statistically significant similarity in distribution of the trajectory volume.

**Figure 10:**
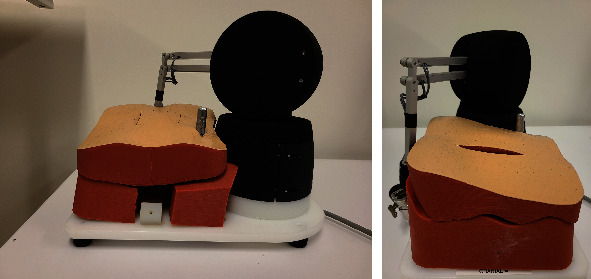
Final design of the mixed reality benchtop configuration (side and front views).

The results from the height changes of the haptic device, as seen in Figures 9(e)–9(g) support the height increase as the users' trajectories were encompassed in the workspace in all configurations. Based on the numerical analyses, the manipulator can move 157 mm along the path of the port while the average of surgeons' path was 142.5 mm. Therefore, this space provided the surgeons with sufficient room to perform the surgical manoeuvres naturally along the path of the port. Based on the data collected from surgeons, the benchtop configuration placed the haptic device on a 25 cm platform higher than the bench model as can be seen in Figure 10. The results for the surgeons showed varying trajectories and volumes, which may be linked to the varying techniques, tools, and motor skills expert surgeons utilize to complete the surgical procedures. The large variations in the trajectory volumes and their encapsulation within the workspace of the haptic device support the use of the haptic device and the mixed reality configuration for a MI spine surgery. The analysis of the trajectory of users with no surgical background but familiarity with the simulator showed that the workspaces of the users may conform as users become more familiar with the simulator and its various components resulting in smoother trajectories which may not necessarily be linked to medical expertise. These smooth trajectories may also be a result of the lack of knowledge on the associated complications with improper technique.

## 4. Conclusions

Haptic feedback is essential for surgical training, especially in MI procedures where the surgeon has limited access to the surgical area. This study presents a numerical method that can be used to determine the configuration of a bench model with a haptic device in a mixed reality simulator where the two components work in complement throughout the simulation. It also demonstrates and evaluates the use of forward and inverse kinematics towards determining the ideal placement of a haptic device in a mixed reality training system. In the present study, the haptic device interacts with the constraints of both bench and virtual models while accommodating the manoeuvrability required to execute the simulated surgery. The determined configuration, using the kinematics of the haptic device and the surgical workspace, was validated using the user trajectory trends. These trends may provide insight into surgeon technique when performing the surgery and their decision-making when interacting with a simulator designed to provide a practice platform for surgeons. This study also puts forth the notion that the enabled surgical workspace offered by simulators should be verified as not to impede the surgical manoeuvers when virtually conducting the operation for training. Often, simulators are studied as a whole when completed versus a component or bottom-up approach as, in part, explored herein. For example, omitting a bottom-up approach may hide isolated system errors, such as restrictive workspace.

Future work would involve the use of automated determination of the benchtop configuration using predefined inputs for the haptic device and surgical workspace, allowing the benchtop to be adjusted to the needs of various procedures and techniques, including different types of spine fusion procedures that are performed on the anatomy modelled on the benchtop.

## Figures and Tables

**Table 1:** The Denavit-Hartenberg (DH) table based on joint parameters of the haptic devices.

Joint	*a*_*i*_ (mm)	*b*_*i*_ (mm)	*α*_*i*_	*θ*_*i*_	Range
1	0	168.6	90°	*θ*_1_	[-96°, 96°]
2	180	0	0	*θ*_2_	[0°, 120°]
3	0	0	90°	*θ*_3_	[-55°, 56.5°]
4	0	168	90°	*θ*_4_	[90°, 270°]
5	7.45	0	0	*θ*_5_	[-80°, 80°]

**Table 2:** Results of the statistical analyses completed for the collected user trajectory and workspace data.

Compared variables	Statistical analysis	Results
Trajectory of experienced surgeons in simulator	∗KWT	*p* *value* ≤ 0.001 *χ*^2^ = 3482.150 At *α* = 0.05
Trajectory volume of users familiar with simulator	∗KWT	*p* *value* = 0.730 *χ*^2^ = 0.620 At *α* = 0.05

∗KWT: Kruskal-Wallis test.

## Data Availability

All relevant data can be found in the results and discussion section of the paper.
